# Preoperative Exclusive Total Parental Nutrition is Associated with Clinical and Laboratory Remission in Severe Active Crohn’s Disease—A Pilot Study

**DOI:** 10.3390/nu12051244

**Published:** 2020-04-28

**Authors:** Eran Zittan, Ian M. Gralnek, Ossama A. Hatoum, Nasser Sakran, Nitzan Kolonimos

**Affiliations:** 1 Ellen and Pinchas Mamber Institute of Gastroenterology and Liver Diseases and the Center for IBD, HaEmek Medical Center, Afula 1834111, Israel; ian_gr@clalit.org.il (I.M.G.); nitzanver@gmail.com (N.K.); 2 Rappaport Faculty of Medicine Technion-Israel Institute of Technology, Haifa 31096, Israel; ossama_ah@clalit.org.il (O.A.H.); sakranas@gmail.com (N.S.); 3 Department of Surgery B, HaEmek Medical Center, Afula 1834111, Israel; 4 Department of Surgery A, HaEmek Medical Center, Afula 1834111, Israel

**Keywords:** total parental nutrition (TPN), crohn’s disease (CD), clinical remission, laboratory remission

## Abstract

Background: The effect of 1–3 months of preoperative exclusive total parental nutrition (TPN) in active Crohn’s disease (CD) patients is not well established. We investigated the efficacy of exclusive TPN in active CD patients. Methods: In a retrospective multi-visit study with data according to our standard care therapy, we assessed clinical and laboratory remission to refractory CD with exclusive preoperative TPN. Inclusion required exclusive preoperative home TPN without additional oral intake for 1–3 months prior to planning surgery. Results: Twenty preoperative CD patients (65% male; 35% female) were on exclusive TPN. The mean age of the cohort was 30.8 ± 11.6 years. Mean duration of preoperative TPN treatment was 73 days (range: 24–142 days). Most patients had terminal ileal (35%) or ileocolonic CD (30%), and with stricturing (B2) phenotype. All 20 patients had significant clinical improvement in all disease activity indices at the end of preoperative TPN (baseline vs. post TPN): HBI 14.5 vs. 4.0 (*p* = 0.001); BMI 19.2 vs. 19.7 kg/m^2^ (*p* = 0.017); CRP 57.2 vs. 10.3 mg/L (*p* = 0.001); Fecal calprotectin (FC) 672 vs. 200 (μg/g); albumin 2.7 vs. 3.6 g/dL (*p* = 0.001). Two patients (10%) no longer required surgery after completion of exclusive TPN. Conclusion: Exclusive preoperative TPN was found to provide significant improvement in nutritional status, and clinical and laboratory remission in severe active Crohn’s patients.

## 1. Introduction

Crohn’s disease (CD) is a chronic relapsing and remitting autoimmune disease marked by transmural inflammation that most commonly affects the small and large intestines. The incidence of inflammatory bowel disease (IBD) continues to increase across newly industrialized societies, with the prevalence of IBD exceeding 0.3% in North America, Europe, and Australia, thus posing a high burden to global health care systems [1].

Although the biologic era with anti-TNF therapy has revolutionized IBD treatment, surgery still plays a crucial role in the management of refractory Crohn’s disease (CD) as a last line therapy, especially in the cases of aggressive penetrating and fibrostenotic phenotypes [2,3]. Approximately 50% of CD patients will require surgery within 10 years of diagnosis, and approximately 25% of those will require a second intestinal resection within 5 years [4,5,6].

In CD patients requiring surgery, adequate nutrition prior to surgery remains a challenge as the degree of malnutrition is affected by the disease severity and duration, and the magnitude of inflammation—which is anorexigenic and thus drives catabolism [7]. Up to 85% of CD surgical candidates are malnourished due to decreased dietary intake and malabsorption as a consequence of their active disease [8]. Moreover, malnutrition is a well-known independent risk factor for postoperative complications, including infection, poor wound healing, and anastomotic leak, that may directly lead to increased costs from increased postoperative hospital length of stay and/or need for reoperation [9,10,11]. In general, postoperative complications occur in approximately 30% of CD patients undergoing intestinal surgery, leading to an increased burden for patients, physicians, and health care systems [12,13]. There are data to support preoperative nutrition optimization to minimize postoperative morbidity in gastrointestinal (GI) surgery [10,14,15]. However, there are limited data to support the use of “exclusive” preoperative total parental nutrition (TPN). The purpose of this pilot study is to assess the efficacy of exclusive TPN on nutritional optimization prior to intestinal surgery in a CD cohort that failed medical management.

## 2. Methods

### 2.1. Study Population

We performed a retrospective cohort study (January 2016 to October 2018) in a single tertiary care IBD center of adult patients (age 18–75 years) with endoscopic and histologically confirmed CD with refractory, complicated disease who were bowel resection candidates. Patients were identified for inclusion in this pilot study by our IBD Unit multidisciplinary team (gastroenterologists, IBD nurse, and dietician). Inclusion criteria for preoperative exclusive TPN therapy were refractory CD patients and at least one of the following indicators for malnutrition according to the Malnutrition Universal Screening Tool (MUST); BMI <18.5 kg/m, weight loss >10% within the last 6 months, and/or serum albumin <3 g/dL. In addition, patients were required to complete at least 3 weeks of exclusive preoperative home TPN without additional oral intake. The TPN intervention was initiated in the in-patient setting and continued with home TPN for the remainder of the therapy duration. We a priori defined refractory CD patients as those with severe clinical, laboratory, radiology (MR-enterography or CT enterography), and malnutrition status. In addition, those patients were non-responsive to at least 3 months of conventional medical therapy prior to TPN therapy and were deemed candidates for definitive surgery such as ileo-colonic resection and/or extensive small bowel resection. Conventional medical therapy was defined as immunomodulatory drugs such as thiopurine /methotrexate and or biologics. Moreover, all patients were on a stable dose and no change of conventional therapy for at least 3 month prior to TPN therapy. No other therapy was added except for TPN.

This study was approved by the institutional research ethics board at Emek Medical Center (0016-16-EMC, date of approval 27 Mar 2016).

### 2.2. Scores

At baseline, the CD location and behavior were recorded based on the modified Montreal classification. Disease location phenotypes include L1 (terminal ileum), L2 (colon), L3 (ileocolon), and L4 (upper GI), which is subdivided into L4a (proximal to the ligament of Treitz) and L4b (distal to the ligament of Treitz). Disease behavior phenotypes include B1 (non-stricturing, non-penetrating), B2 (stricturing/stenotic), B3 (penetrating), and P (perianal disease modifier). We also prospectively collected CD severity data using the Harvey–Bradshaw Index (HBI). The HBI is a clinical assessment of Crohn’s disease activity based on five clinical parameters: (1) patient well-being, (2) abdominal pain, (3) number of liquid or soft stools, (4) abdominal mass, and (5) complications. A cumulative score <5 suggests remission, a score between 5 to 7 suggests mild activity, a score between 8 to 16 suggests moderate activity, and a score >16 suggests severe disease activity.

C-reactive protein (CRP) is an indicator of systemic inflammation and can be a useful marker to monitor the effect of treatment on systemic inflammation. The laboratory at our institution used a cutoff value >5 mg/L to define abnormally increased CRP levels.

Fecal calprotectin (FC) is a noninvasive stool biomarker to monitor intestinal inflammation in patients with CD [16,17,18,19,20]. The laboratory at our institution used FC high range immunoassay kit (0–2000 μg/g). We used FC for a subset of our cohort (10/20) as an additional noninvasive stool biomarker.

Albumin is a protein synthesized by the liver that can be used as a surrogate marker of nutrition to help assess protein intake. Moreover, albumin level is influenced by the catabolic status of the patient and inflammation. An albumin range between 3.4 to 5.4 g/dL was considered normal by the laboratory at our institution.

The body mass index (BMI) is a weight-to-height ratio to determine if a patient is at a healthy weight. A BMI score <18.5 is considered underweight, a score between 18.5 to 24.9 is normal, a score between 25 to 29.9 is overweight, and a score >30 is obese. The HBI, BMI, and albumin were recorded at baseline and after completion of the exclusive preoperative TPN intervention.

### 2.3. Total Parental Nutrition (TPN)

For all patients we used standardized formulations of TPN. First, we admitted to hospital (1–3 days) all patients for PICC line venous access insertion and initiation of TPN before discharge to home TPN. No prophylactic antibiotic were used as per our protocol. Home TPN was managed per sterile protocol. Second, all patients were instructed and followed by our dedicated IBD dietitian during the home TPN period. Close follow-up (every 1–2 weeks clinical visit with our multidisciplinary team), included monitoring for side effects, TPN compliance, and no oral intake.

### 2.4. Statistics

Statistical analyses were performed using SPSS version 24.0 software (IBM Corporation, Armonk, NY, USA). Demographic and clinical parameters were evaluated with means and standard deviations or medians and interquartile ranges as indicated. The nonparametric Mann–Whitney U test for comparison of two groups was performed to compare subgroups as variables were not normally distributed.

## 3. Results

Twenty preoperative patients (65% male; 35% female) with refractory CD on exclusive TPN were included (Table 1). The mean age of the patients was 30.8 ± 11.6 years, and the median disease duration was 8 years (IQR 2.5–11). Mean duration of TPN treatment was 73 ± 28 days, and every patient completed at least 24 days of exclusive TPN (range 24–142 days).

The most common disease locations in our cohort included the terminal ileum (35%), ileocolonic region (30%), and a combination of the terminal ileum plus the more proximal small intestine distal to the ligament of Treitz (25%). One patient had CD localized to the colon, while another patient had a combination of colonic CD with small intestinal involvement distal to the ligament of Treitz excluding the terminal ileum (Table 1).

Disease behavior of the study population is summarized in Table 1. Nine patients (45%) had B2 stricturing/stenotic phenotype, which was the most common disease pattern in the study. Nineteen patients (95%) had some degree of structuring, including 20% with penetrating lesions (B3) and 15% with non-penetrating lesions (B1). Perianal disease (P) was present in 20% of patients.

**Figure 1:**
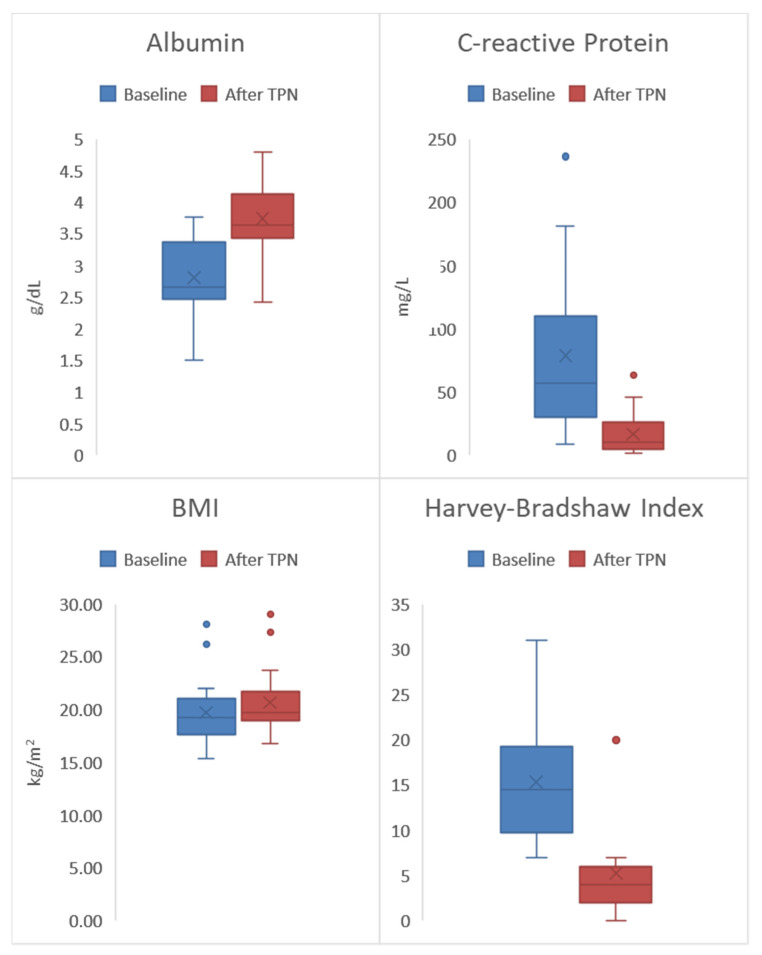
All 20 patients had significant clinical improvement in all disease activity indices at the end of preoperative exclusive total parental nutrition (TPN) (baseline vs. post TPN): Harvey–Bradshaw Index (HBI) 14.5 vs. 4.0 (*p* = 0.001); Body mass index (BMI) 19.2 vs. 19.7 kg/m^2^ (*p* = 0.017); C-reactive protein (CRP) 57.2 vs. 10.3 mg/L (*p* = 0.001); Albumin 2.7 vs. 3.6 (g/dL) (*p* = 0.001).

All 20 patients in the study demonstrated significant clinical, laboratory, and radiographic improvement in all disease activity and nutritional indices from baseline to the end of preoperative exclusive TPN treatment (Table 2, Figure 1). In our cohort, all patients had significant laboratory remission including decrease in white blood count and platelet count to normal range, as well as increased hemoglobin level to the normal range. We also confirmed significant radiographic improvement (significant decrease in mucosal enhancement per MR Enterography/CT enterography) after TPN therapy.

Overall, there was a significant increase in albumin from a median of 2.7 g/dL to 3.6 g/dL (*p* = 0.001). These results suggest that overall the study population began with inadequate protein intake at baseline—indicated by a low median albumin score (<3.4 g/dL)—and that protein intake significantly improved by the end of the TPN treatment due to the normal post-treatment albumin level of 3.6 g/dL. The laboratory marker for systemic inflammation CRP, significantly decreased from a median of 57.2 mg/L to 10.3 mg/L (*p* = 0.001). Fecal calprotectin significantly decreased from a median of 679 (μg/g) to 200 (μg/g) (*p* = 0.001). Significant increases in weight gain were also observed by an overall increase in the median BMI from 19.2 kg/m^2^ to 19.7 kg/m^2^ (*p* = 0.017). Finally, overall clinical improvement in the study population was demonstrated by a significant decrease in the median HBI from 14.5 to 4.0 (*p* = 0.001). This represents a significant decrease in clinical disease activity from moderate disease (HBI score 8–16) to clinical remission (HBI score <5). Of note, two patients (10%) no longer required bowel resection after the completion of exclusive TPN due to significant clinical and laboratory remission with concurrent alleviation of CD symptoms.

## 4. Discussion

Based on our study, preoperative exclusive TPN was found to lead to statistically significant weight gain, decreased inflammatory biomarkers (FC, CRP) and improved clinical disease activity and nutrition. The observed significant improvement in the nutritional status in patients with active moderate-to-severe CD on TPN has been previously reported. The BMI increase of 0.5 kg/m^2^ in our study was similarly found in a previous study by Seo et al., which demonstrated a statistically significant BMI increase of 0.4 kg/m^2^ (*p* < 0.01) in Crohn’s disease patients who received 3 weeks of TPN [21]. Furthermore, several other studies have similarly demonstrated a significant elevation and normalization of serum albumin levels after receiving TPN therapy [22,23,24]. However, to our knowledge, no prior study has prospectively evaluated the efficacy of home exclusive TPN using objective indices of both nutrition and disease activity. The robustness of prospectively collected data on BMI, serum albumin, serum CRP, FC, and the HBI is a major strength of our study. In addition, our team comprised a specially trained IBD dietician who administered the home TPN and accurately recorded weight and nutrition related information on each patient in our database.

Although none of the participants in this study developed complications secondary to the home-exclusive TPN intervention, the literature reports complication rates as high as 61% [22,25,26]. This includes a prospective study from the Mayo Clinic that reported a 27% infection rate in IBD patients receiving home TPN for less than 6 months and a 47% infection rate in those receiving home TPN for more than 6 months [25]. In addition to catheter-associated infection/sepsis, other reported complications included blocked or damaged catheters, venous thrombosis, liver failure, dehydration, and electrolyte imbalances [26,27].

Although the results of this present study are encouraging in improving the preoperative nutritional status and Crohn’s disease status, there are several limitations. First, both the patients and researchers were unblinded, which has the potential to introduce bias due to the expectations of disease and nutritional improvement from exclusive TPN treatment. Second, the small sample size (*n* = 20) limits the statistical power and generalization of results of this study. A future larger, similar study is warranted to further validate these results. Furthermore, an exclusive home TPN regimen of 1–3 months duration is very challenging for the patients to strictly adhere to and is nearly impossible for the clinical staff to guarantee compliance of the in-home treatment (e.g., absolutely no oral intake in addition to TPN). Additionally, home TPN is expensive. The estimated cost of home TPN was $280 ($238–390) per day depending on the glucose and lipid content [28]. The high cost of home TPN may be prohibitive to those without health insurance.

Future aims will be to assess the postoperative outcomes of patients preoperatively treated with exclusive TPN matched to standard-of-care controls (i.e., no preoperative exclusive TPN). We hypothesize that patients treated with preoperative exclusive TPN will have improved perioperative outcomes with decreased infections and anastomotic leaks due to a reduction in the preoperative inflammatory burden combined with an improved nutritional status.

In summary, exclusive preoperative TPN was associated with significant weight gain, decreased inflammatory biomarkers, improved nutritional status, and improved clinical disease activity as measured by HBI. These data suggest that at least 3 weeks of exclusive TPN are associated with significant improvements in both clinical and laboratory indicators of CD remission.

## Figures and Tables

**Table 1:** Demographics and disease characteristics.

Age (years)	30.8 ± 11.6
Male	13 (65%)
Female	7 (35%)
Disease Duration (years)	8 (IQR 2.5–11)
BMI (kg/m^2^)	19.2 (IQR 17.7–21)
Albumin (g/dL)	2.7 (IQR 2.5–3.4)
CRP (mg/L)	57.2 (IQR 30.4–110)
Fecal Calprotectin (μg/g)	679 (IQR 332–924)
Disease Location:	
L1	7 (35%)
L2	1 (5%)
L3	6 (30%)
L1, L4b	5 (25%)
L3, L4b	1 (5%)
Disease Behavior:	
B2	9 (45%)
B2, B3	3 (15%)
B1, B2	4 (20%)
B2, P	1 (5%)
B3, P	1 (5%)
B1, B2, P	1 (5%)
B2, B3, P	1 (5%)

Body mass index (BMI); C-reactive protein (CRP); L1 = terminal ileum; L2 = colon; L3 = ileocolonic; L4b = upper GI distal to ligament of Treitz excluding terminal ileum; B1 = nonstricturing, nonpenetrating; B2 = stricturing/stenotic; B3 = penetrating; P = perianal disease modifier.

**Table 2:** Outcomes.

Assessment	Baseline	After TPN Treatment	*p*-Value
Albumin (g/dL)	2.7 (IQR 2.5–3.4)	3.6 (IQR 3.4–4.1)	0.001
CRP (mg/L)	57.2 (IQR 30.4–110)	10.3 (IQR 5–26.7)	0.001
Fecal Calprotectin (μg/g)	679 (IQR 332–924)	200 (IQR 127–278)	0.001
BMI (kg/m^2^)	19.2 (IQR 17.7–21)	19.7 (IQR 18.9–21.8)	0.017
HBI	14.5 (IQR 9.7–19.2)	4 (IQR 2–6)	0.001
